# Analysis of *Kudoa septempunctata* as a cause of foodborne illness and its associated differential diagnosis

**DOI:** 10.4178/epih.e2017014

**Published:** 2017-03-31

**Authors:** Sung Uk Lee

**Affiliations:** Jeju Special Self-Governing Provincial Office, Jeju, Korea

**Keywords:** Food parasitology, Myxozoa, *kudoa septempunctata*, *Staphylococcus aureus*, *Bacillus cereus*, Korea

## Abstract

**OBJECTIVES:**

Recently *kudoa septempuctata* in olive flounders is suggested as a cause of food poisoning, however whether *kudoa septempuctata* can affect human gastrointestinal systems is controversial and its pathogenecity remains unclear. In view of the field epidemiology, food poisonings caused by *kudoa septempuctata* should be distinguished from those caused by *Staphylococcus aureus* and *bacillus cereus*.

**METHODS:**

The statistics of food poisoning investigations published by Korea Centers for Disease Control and Prevention in 2013-2015 were reviewed. The characteristics of *kudoa septempuctata* food poisoning reported by Korea Centers for Disease Control and Prevention were reviewed. Information regarding clinical symptoms or epidemiology was extracted.

**RESULTS:**

Total eleven *kudoa septempuctata* food poisoning cases were analyzed. Food poisonings caused by *kudoa septempuctata*, *Staphylococcus aureus* and *bacillus cereus* have clinical and epidemiological similarities. Forty five percent of food poisoning outbreaks occurred in Korea was concluded as unknown. The food poisoning caused by *Staphylococcus aureus* and *bacillus cereus* accounted for 4.5% (50/1,092) of all food poisoning outbreaks in Korea between 2013 and 2015.

**CONCLUSIONS:**

This study suggests the possibilities of misdiagnosis in the investigations of food poisoning by *Staphylococcus aureus* and bacillus cereus with *kudoa septempuctata*.

## INTRODUCTION

Foodborne outbreaks caused by *kudoa septempunctata* and the characteristics of these cases were reported by Korea Centers for Disease Control and Prevention (KCDC) in its annual surveillance report. In addition, KCDC suggests the need for a new nationwide policy to prevent the spread of *K. septempunctata* and consequent food poisoning [1]. The numerous myxosporean species of the genus *Kudoa* reported around the world are not pathogenic to humans [2]. However, *K. septempunctata* has recently been considered as a human pathogen since it was first identified in olive flounders. This parasite was identified as a causative agent of food poisoning by the Ministry of Health, Labour and Welfare in Japan in December 2012, and its pathogenesis was also studied in an experiment using 4 to 5-day old mice [3,4]. However, other mice studies had conflicting results [5], and studies on humans have not been reported with regard to this new species. Based on these grounds, Chung et al. [6] recently suggested that epidemiologic causality between *K. septempunctata* and food poisoning lacks scientific validity.

Meanwhile, based on the epidemiological investigation data, the cases of *K. septempunctata* food poisoning are characterized by a short incubation period and mild gastrointestinal distress [4], which are similar to the epidemiologic and clinical features of *Staphylococcus aureus* and *Bacillus cereus food poisoning*. Therefore, a differential diagnosis of *K. septempunctata* and the aforementioned bacteria is critical in identifying the former as a new foodborne pathogen. In this study, cases of *K. septempunctata* outbreak were investigated and compared with those caused by *S. aureus* and *B. cereus*. Moreover, the causes of foodborne illnesses that occurred from 2013 to 2015 were also analyzed.

## MATERIALS AND METHODS

*K. septempunctata* outbreaks that were reported in the 2015 Annual Infectious Disease Surveillance Report and its appendix were reviewed to determine the characteristics of *K. septempunctata* food poisoning [1].

The identified characteristics were then compared with those of *S. aureus* (Chapter 8) and *B. cereus* (Chapter 1) outbreaks, which were extracted from the book “*Pathogens and Toxins in Food*” [10]. Individual *K. septempunctata* cases described in the appendix were analyzed to determine epidemiological features and current trends in epidemiologic investigations. To identify the causes of foodborne diseases in Korea, the incidence rates of water and foodborne diseases were reviewed in the aforementioned report of KCDC from 2013 to 2015 [7-9]. Therefore, outbreaks with unknown causes and *S. aureus* and *B. cereus* outbreaks, each of which was presented as a percentage ratio to the total outbreaks, will be identified.

## RESULTS

### Comparison of clinical and epidemiological characteristics of *K. septempunctata* and *S. aureus* and *B. cereus* cases

*S. aureus* produces heat-resistant enterotoxins, and *B. cereus* forms heat-resistant spores. These products are considered as the main cause of food poisoning (Table 1) [10]. Although cooking can kill these causal pathogens, toxins and spores are not destroyed and can cause food poisoning. According to a study about *K. septempunctata* with respect to human intestinal cells, this parasite causes food poisoning when its sporoplasm invades the intestinal cells. Although *S. aureus* and *B. cereus* are common foodborne pathogens, the number of outbreaks caused by these bacteria is underestimated because the symptoms of mild food poisoning are not reported worldwide. In addition, these pathogens are difficult to detect through *in vitro* diagnostic tests during epidemiologic investigations [11,12]. The ratio of *K. septempunctata* outbreaks to total foodborne illness outbreaks has not been clearly identified yet. *S. aureus* and *B. cereus* actively grow at a high temperature (20- 37°C), causing a higher incidence of infections in summer than in winter. However, no incidence of *K. septempunctata* food poisoning in the months of June and July was reported, while the incidence was spread evenly across the other months of the year (5-15 cases per month), showing the highest incidence in May. The KCDC identified a possible association between this incidence pattern and changes in sea water temperature [1]. Nonetheless, Song et al. [2] stated that *K. septempunctata* is detected in olive flounders throughout the year without changes in June and July. Their finding confirms that the incidence of food poisoning is more likely related to the consumption of olive flounders, which significantly decreases in summer. With the mean incubation period of less than six hours, the three pathogens mainly caused diarrhea and vomiting, and all symptoms lasted for less than 24 hours, with similar characteristics. *S. aureus* colonizes the human skin and nasal mucous membranes, whereas *B. cereus* is widely distributed in the natural environment, such as soil. Although the life cycle of *K. septempunctata* is not completely known, it is a parasite commonly found in olive flounders [13]. *S. aureus* and *B. cereus* can cause food poising through a variety of ways, including the ingestion of contaminated meat and dairy products and undercooked rice. By far, *K. septempunctata* infection only occurred after the consumption of olive flounder.

### Review of *K. septempunctata* outbreaks

A total of 11 *K. septempunctata* outbreaks that occurred in 2015 are summarized (Table 2). Two out of 11 cases were caused by the ingestion of raw olive flounder, whereas the remaining nine cases were associated with the consumption of various fish, including olive flounder, shellfish, and sushi. Diarrhea was a more common symptom than vomiting in six outbreaks. However, vomiting was common in five outbreaks. The average incubation period ranged from 2 to 10 hours. Based on the results of laboratory tests, *K. septempunctata* was found in patients of 10 outbreaks and in official food samples in the remaining one outbreak (Outbreak 9) in which patients were not infected. In Outbreak 5, *K. septempunctata* was found along with *B. cereus* in samples of patients, and poor sanitation was observed. In Outbreak 11, *K. septempunctata* was detected in two patients and in one asymptomatic individual.

### Common causative agents of foodborne illness

The causes of water and foodborne illnesses that occurred from 2013 to 2015 were analyzed (Table 3). The causative agent was unknown for 36 to 51% of the total outbreaks during the 3-year period, and the source of infection (food or drinking water) was unknown for 75 to 81% of the total outbreaks in 2013 and 2014 (2015 data not available). Norovirus, which is the most common gastrointestinal infection source in winter, was the leading cause of gastrointestinal infection in our review, accounting for between 16 and 19% of the total cases. Norovirus was followed by patho - genic intestinal bacteria (6-10%)—*Clostridium perfringens* (3-11%), and Salmonella spp. (2-7%). A total of 26 *S. aureus* outbreaks oc - curred during the 3-year period, accounting for 2.4% of the total foodborne illness outbreaks (26/1,092), and *B. cereus* outbreaks constituted 2.2% (24/1,092). In 2015 alone, *K. septempunctata* is the fifth most common cause of food poisoning with 11 outbreaks, accounting for 2.6% of the total outbreaks.

## DISCUSSION

*K. septempunctata* has emerged as a foodborne pathogen that is associated with the consumption of olive flounder, and the national epidemiologic investigation report on foodborne illness recommends tests for *K. septempunctata* infection to those who eat olive flounder. Song et al. [2] reported that *K. septempunctata* was found in 4.9% of cultured olive flounder that were investigated. Given that olive flounder is a highly favored fish in Korea, a substantial amount of olive flounder parasitized with *K. septempunctata* is consumed. *K. septempunctata* infection is observed in cultured olive flounders that are distributed and remained intact, regardless of preparation methods, until it is eaten. The parasitic infection needs to be prevented during fish farming. As *K. septempunctata* has been discovered in olive flounder only, the epidemiologic investigation of food poisoning focused on detecting *K. septempunctata* DNA from clinical samples of patients who consumed olive flounders less than 24 hours before testing, regardless of the test results of food and environment samples. Thus, the test approach differs from other methods of foodborne illness investigation.

However, such an approach does not seem complete amid uncertainty over the pathogenic effects of *K. septempunctata* on food poisoning. Its pathogenicity to humans has been difficult to verify, and the animal-based studies revealed contradicting results, as discussed by Chung et al. [6]. The results of the pathogenic identification method that focused on *K. septempunctata* in the clinical samples of patients also contradict the findings in the study conducted by Ahn et al. [14] where *K. septempunctata* DNA was detected in mice without gastrointestinal symptoms. Therefore, based on these considerations, *K. septempunctata* may exist in fecal samples of patients who consumed olive flounders infected with the parasite, regardless of the development of food poisoning. As mentioned in Results, *K. septempunctata* was detected in a healthy individual in the control group in one out of the 11 outbreaks. The examination for the presence of *K. septempunctata* DNA in patients does not confirm the parasitic contribution to the onset of food poisoning.

Then, why does food poisoning occur after the consumption of olive flounder infected with *K. septempunctata?* Authors suggest the possibility that food poisoning caused by *S. aureus* or *B. cereus* is mistaken as *K. septempunctata* food poisoning. As discussed in Results, the cases of food poisoning caused by these three pathogens share similar epidemiological and clinical features, except that *K. septempunctata* is associated with the consumption of raw olive flounder. As mentioned in Results, *S. aureus* and *B. cereus* are the most common cause of food poisoning. However, each of these bacteria accounted for approximately 2% of the total foodborne disease outbreaks that occurred during the 3-year period. In 2015, the outbreaks of *S. aureus* and *B. cereus* food poisoning were lower than those of *K. septempunctata* food poisoning. Overall, the cause was unknown for half of all outbreaks, suggesting the possibility that bacterial food poisoning was not properly verified in numerous cases and that the actual outbreaks of these two bacteria were higher than that reported. Moreover, some *S. aureus* and *B. cereus* outbreaks that were classified as unknown causes because of the above-mentioned reasons can be mistaken as *K. septempunctata* outbreaks, given that similar outcomes were observed. Among the 11 outbreaks described in Results, one *K. septempunctata* case and one *B. cereus* case were found in Outbreak 5. If the pathogenicity of the former is considered unclear, Outbreak 5 is suggested as a *B. cereus* case.

However, *S. aureus* and *B. cereus* are not easily identified as the causative agents of food poisoning, when compared with other food poisoning pathogens. To verify the causal relationship between the two bacteria and food poisoning with epidemiological data, the growth of epidemiologically relevant food-borne bacteria or enterotoxins and the clinical features (a short incubation period and mild gastrointestinal distress) of foodborne intoxication are important. Even in cases where foodborne intoxication is suspected based on clinical features, the availability of official food samples and the conditions for stool culture are significantly low, making in vitro diagnosis possible in only limited cases. Therefore, most cases are classified as unknown causes [10]. In cases where the food that caused food poisoning is recooked and distributed, in vitro diagnostic tests were then difficult to perform because cooking kills causative agents and the heat-resistant enterotoxins and spores that cause food poisoning.

During epidemiologic investigations, the identification of causes of food poisoning outbreaks should be carried out in a discreet manner because the involved parties, such as food supplier and future public health policy, may be affected. To further recognize the pathogenic effect of *K. septempunctata* on food poisoning, consistent effort is needed to improve the identification rate of *S. aureus* and *B. cereus* food poisoning cases, and further studies on the pathogenesis of *K. septempunctata* should be conducted.

## Figures and Tables

**Table 1.** Comparison of clinical and epidemiological characteristics between *K. septumpunctata*, *S. aureus*, and *B. cereus* food poisonings

	*K. septumpunctata*	*S. aureus*^1^	*B. cereus*^1^
Pathogenesis	Invasion of intestinal epithelium by sporoplasm (*in vitro*)	Heat-resistant enterotoxin	Heat-resistant spore
Proportion of food borne illness	Not assessed	1-5%, usually underestimated	1-30%, usually underestimated
Seasonal variation	Peak in May, but reduces in summer season	May be more in warm-hot season than cold, rapid growth at room air	May be more in warm-hot season than cold, rapid growth at room temperature
Main clinical symptoms	Diarrhea, vomiting, abdominal pain	Diarrhea, vomiting, abdominal pain	Diarrhea, vomiting, abdominal pain
Incubation periods (hr)	Average 2 (1-15)	0.5-8.0	Vomiting: 1-6
			Diarrhea: 6-24
Duration and severity of illness	Usually resolved within 24 hr, self limiting	Usually resolved within 24 hr, self limiting	Usually resolved within 24 hr, self limiting
Life cycle	Unknown	Human skin, nasal passage, hair	Soil
Common source of infection	Intake of raw olive flounder	Meat, poultry, egg and dairy product, salads	Rice, meat, soup

1 Characteristics of S. aureus and B. cereus referred from Juneja et al. Pathogens and toxins in foods: challenges and interventions. Washington, DC: ASM Press; 2010 [10].

**Table 2.** Summary of cases of food poisoning caused by *K. septempunctata*

Case	I	II	III	IV	V	VI	VII	VIII	IX	X	XI	
Population size (n)	8	13	4	4	27	12	11	18	10	9	38
No. of patients	7	13	3	4	13	8	9	12	9	6	8
Causative food	Sashimi set	Sashimi with olive flounder and sushi, soup	Sashimi with olive flounder and shellfish	Sashimi with olive flounder	Sashimi set with various fish and shellfish	Sashimi set and sushi, beef tartare	Sashimi with olive flounder	Buffet including sushi	Sashimi set and sushi	Sashimi set	Mix of sauced sashimi and vegetables
Symptoms	D〉V	V>D	D〉V	D>V	V>D	V>D	D〉V	V>D	D>V	V>D	D〉V
Incubation period, average (hr)	5.8	4.0	3.5	4.5	6.0	3.8	10.0	4.1	3.6	5.0	2.0
Laboratory test											
Food	(-)	*Kudoa*	(-)	(-)	(-)	*Kudoa*	(-)	(-)	*Kudoa*	*Kudoa*	(-)
Environment	(-)	(-)	(-)	(-)	Problem in sanitation	(-)	(-)	(-)	(-)	(-)	(-)
Food preparer	(-)	(-)	(-)	(-)	(-)	(-)	(-)	(-)	(-)	(-)	(-)
Patient (no. of positive)	*Kudoa* (6)	*Kudoa* (8)	*Kudoa* (1)	*Kudoa* (2)	*Kudoa* (1)	*Kudoa* (1)	*Kudoa* (2)	*Kudoa* (3)	(-)	*Kudoa* (6)	*Kudoa* (2), but *Kudoa* in healthy (1)
					*B. cereus* (1)						

V, vomiting; D, diarrhea.

**Table 3.** Annual trends in cause of food poisoning^1^

Rank	2015		2014		2013		
	Total	422	Total	409	Total	261
	Unknown	215 (51)	Unknown	186 (45)	Unknown	94 (36)
1	Norovirus	79(19)	Norovirus	48(12)	Norovirus	42(16)
2	*E. coll*	26 (6)	*E. coll*	41 (10)	*C. perfrlngens*	30(11)
3	*Campylobacter*	22 (5)	*C. perfrlngens*	29 (7)	*E. coll*	19 (7)
4	*C. perfrlngens*	14 (3)	Salmonellosis	28 (7)	Salmonellosis	12 (5)
5	Salmonellosis	10 (2)	*Campylobacter*	23 (6)	*Shigella spp.*	8 (3)
6	*S. aureus*	8 (2)	*S. aureus*	13 (3)	*B. cereus*	8 (3)
7	*B. cereus*	6 (1)	*B. cereus*	10 (2)	*V. parahaemolytlcus*	8 (3)
8	*V. parahaemolytlcus*	3(1)	*V. parahaemolytlcus*	6(1)	*S. aureus*	5(2)

Values are presented as number (%).

1 Data from Korea Centers for Disease Control and Prevention. Epidemiological investigation of infectious diseases in Korea, annual report from 2013 to 2015 [7-9].
