# Supplementary material for: Analysis of Kudoa septempunctata as a cause of foodborne illness and its associated differential diagnosis
Source: Epidemiol Health. 2017 Mar 31;39:e2017014. doi: 10.4178/epih.e2017014 (PMC5543295; doi:10.4178/epih.e2017014)
Supplement: Supplementary file 1 [file epih-39-e2017014-supplementary.pdf]

Open Access

Volume: 39, Article ID: e2017014, 5 pages
https://doi.org/10.4178/epih.e2017014

epiH Epidemiology and Health

EPIDEMIOLOGIC INVESTIGATION

식중독 원인으로서의 칠성 쿠도아충과 감별진단

이성욱

제주도청 보건위생과

Analysis of *Kudoa septempunctata* as a cause of foodborne illness and its associated differential diagnosis

Sung Uk Lee

Jeju Special Self-Governing Provincial Office, Jeju, Korea

KOREAN SUMMARY

최근 광어 섭취 후 발생한 식중독의 한 원인으로 쿠도아충이 지목되고 있다. 하지만 쿠도아충에 의한 식중독은 인체 감염에 대한 근거가 부족하고, 황색포도알균이나 바실루스 세레우스 식중독과 감별이 어려워 진단에 있어 주의를 요한다.

Objectives: Recently *kudoa septempuctata* in olive flounders is suggested as a cause of food poisoning, however whether *kudoa septempuctata* can affect human gastrointestinal systems is controversial and its pathogenecity remains unclear. In view of the field epidemiology, food poisonings caused by *kudoa septempuctata* should be distinguished from those caused by *staphylococcus aureus* and *bacillus cereus*.
Methods: The statistics of food poisoning investigations published by Korea Centers for Disease Control and Prevention in 2013-2015 were reviewed. The characteristics of *kudoa septempuctata* food poisoning reported by Korea Centers for Disease Control and Prevention were reviewed. Information regarding clinical symptoms or epidemiology was extracted.
Results: Total eleven *kudoa septempuctata* food poisoning cases were analyzed. Food poisonings caused by *kudoa septempuctata*, *staphylococcus aureus* and *bacillus cereus* have clinical and epidemiological similarities. Forty five percent of food poisoning outbreaks occurred in Korea was concluded as unknown. The food poisoning caused by *staphylococcus aureus* and *bacillus cereus* accounted for 4.5% (50/1,092) of all food poisoning outbreaks in Korea between 2013 and 2015.
Conclusions: This study suggests the possibilities of misdiagnosis in the investigations of food poisoning by *staphylococcus aureus* and *bacillus cereus* with *kudoa septempuctata*.

KEY WORDS: Food parasitology, Myxozoa, *Kudoa septempunctata, Staphylococcus aureus, Bacillus cereus*, Korea

Correspondence: Sung Uk Lee
Jeju Special Self-Governing Provincial Office, 6 Munyeon-ro, Jeju 63122, Korea
E-mail: sulee86rad@gmail.com

Received: Mar 23, 2017 / Accepted: Mar 31, 2017 / Published: Mar 31, 2017

This article is available from: http://e-epih.org/

This is an open-access article distributed under the terms of the Creative Commons Attribution License (http://creativecommons.org/licenses/by/4.0/), which permits unrestricted use, distribution, and reproduction in any medium, provided the original work is properly cited.

© 2017, Korean Society of Epidemiology

서 론

한국질병관리본부에서 발간한 2015년 감염병역학조사연보에서는 칠성쿠도아충에 의한 식중독의 사례조사 및 특성을 보고하고, 칠성쿠도아충으로 인한 식중독 예방을 위한 범국가적 정책의 필요성에 대하여 제안하였다[1]. 쿠도아속 점액포자충은 전세계적으로 수많은 종류가 보고 되어 오고 인체에 대한 병원성이 없는 것으로 생각되어 왔지만[2], 쿠도아속 중 최초로 광어에서 발견되는 칠성쿠도아충의 인체에 대한 병원성이 최근 제기되었다. 칠성쿠도아충은 2012년 12월 일본 보건후생성에 의해 식중독

원인 병원체로 지목되었고, 연령이 4-5일인 ddY mice를 이용한 동물 실험을 근거로 병인성이 주장되었다[3,4]. 하지만 타연구자에 의한 mice를 이용한 동일한 실험에서 정반대의 결과가 보고되었고[5], 인체에 대한 임상연구는 보고된 적이 없어, 최근 Chung et al. [6]은 칠성쿠도아충과 식중독 간의 역학적 인과성의 과학적 타당성이 떨어짐을 주장하였다.

한편 식중독 역학조사의 관점에서 칠성쿠도아충에 의한 것으로 추정되는 식중독사례들은 짧은 잠복기와 경미한 소화기계 증상을 특징으로 하는데[4], 이는 독소형 식중독(황색포도알균증 또는 바실루스 세레우스)의 식중독의 역학적 특성 및 임상적 증상과 유사하다. 칠성쿠도아충을 식중독의 새로운 병원체로 지정하기 위해선 위 두 식중독병원체와 감별하는 것이 중요하다. 이에 저자는 먼저 쿠도아충 식중독의 개별 사례를 살펴보고, 쿠도아충 식중독과 황색포도알균과 바실루스 세레우스로 인한 식중독을 비교해보겠다. 마지막으로 최근 3년간의 식중독 원인병원체 규명 현황을 살펴보겠다.

연구방법

칠성쿠도아충 식중독 사례의 정확한 특성을 파악하기 위해 2015년도 감염병역학조사연보에 수록된 칠성쿠도아충의 식중독 사례보고 및 부록이 검토되었다[1].

사례보고에 기술된 칠성쿠도아충의 특성과 “Pathogens and Toxins in Food [10]”에 기술된 황색포도알균(Chapter 8)과 바실루스 세레우스(Chapter 1) 식중독 특성을 비교하였다. 또한 부록에 포함된 쿠도아충 식중독 개별 사례 분석을 통해 칠성쿠도아충 식중독의 역학조사 현황 및 특성에 대하여 파악하였다. 한국의 식중독 사례 규명 현황 분석을 위해 한국질병관리본부에서 발간한 2013-2015년 감염병역학조사연보에 포함된 연도별 수인성 및 식품매개질환 발생 현황이 검토되었다[7-9]. 식중독 원인 불명률과 황색포도알균과 바실루스 세레우스 식중독의 규명률이 각각 파악되었다.

연구결과

1. 칠성쿠도아충, 황색포도알균과 바실루스 세레우스 식중독 특성 비교

황색포도알균은 열저항성이 있는 장독소를 생성하고 바실루스 세레우스는 열저항성이 있는 포자가 식중독을 일으키는 것으로 알려져 있다(Table 1)[10]. 이 두 병원체는 음식제공 전 재가열하게 되면 원인병원체는 열에 의해 사라지지만 식중독을 일으키는 독소와 포자는 여전히 음식에 남아 있는다. 칠성쿠도아충은 인체장세포를 이용한 실험에서 포자원형질이 장세포를 침범하여 식중독을 일으키는 것으로 추정하였다. 황색포도알균과 바실루스 세레우스는 가장 흔한 식중독 원인들이지만 전 세계적으로 식중독 증상의 가벼움으로 인한 미신고와 역학조사 시 실험실적 병원체 검출의 어려움 등으로 인하여 통상적으로 전체 발생률이 실제 발생률에 비해 매우 저평가되는 식중독 원인들로 알려져 있다[11, 12]. 아직 칠성쿠도아충이 전체 식중독에서 차지하는 비중은 제대로 파악되지 않았다. 황색포도알균과 바실루스 세레우스는 상온(20-37°C)에서 가장 활성화되고, 겨울보다는 기온이 따뜻해질수록 발생률이 증가한다. 칠성 쿠도아충은 연중 비슷한 수준 (5-15명/월)으로 식중독을 일으키나 5월에 특히 증가하고 6, 7월은 전혀 보고되지 않았다. 질병관리본부에서는 이를 수온의 변화에 따른 영향으로 추측하였지만[1], Song et al. [2]에 따르면 광어에서 칠성쿠도아충이 6, 7월에 검출률이 떨어지는 것은 아니고 1월부

Table 1. Comparison of clinical and epidemiological characteristics between *K. septumpunctata*, *S. aureus*, and *B. cereus* food poisonings

	K. septumpunctata	*S. aureus*[1]	*B. cereus*[1]
Pathogenesis	Invasion of intestinal epithelium by sporoplasm (*in vitro*)	Heat-resistant enterotoxin	Heat-resistant spore
Proportion of food borne illness	Not assessed	1-5%, usually underestimated	1-30%, usually underestimated
Seasonal variation	Peak in May, but reduces in summer season	May be more in warm-hot season than cold, rapid growth at room air	May be more in warm-hot season than cold, rapid growth at room temperature
Main clinical symptoms	Diarrhea, vomiting, abdominal pain	Diarrhea, vomiting, abdominal pain	Diarrhea, vomiting, abdominal pain
Incubation periods (hr)	Average 2 (1-15)	0.5-8.0	Vomiting: 1-6<br>Diarrhea: 6-24
Duration and severity of illness	Usually resolved within 24 hr, self-limiting	Usually resolved within 24 hr, self-limiting	Usually resolved within 24 hr, self-limiting
Life cycle	Unknown	Human skin, nasal passage, hair	Soil
Common source of infection	Intake of raw olive flounder	Meat, poultry, egg and dairy product, salads	Rice, meat, soup

[1]Characteristics of *S. aureus* and *B. cereus* referred from Juneja et al. Pathogens and toxins in foods: challenges and interventions. Washington, DC: ASM Press; 2010 [10].

Table 2. Summary of cases of food poisoning caused by *K. septempunctata*

Case	I	II	III	IV	V	VI	VII	VIII	IX	X	XI
Population size (n)	8	13	4	4	27	12	11	18	10	9	38
No. of patients	7	13	3	4	13	8	9	12	9	6	8
Causative food	Sashimi set	Sashimi with olive flounder and sushi, soup	Sashimi with olive flounder and shellfish	Sashimi with olive flounder	Sashimi set with various fish and shellfish	Sashimi set and sushi, beef tartare	Sashimi with olive flounder	Buffet including sushi	Sashimi set and sushi	Sashimi set	Mix of sauced sashimi and vegetables
Symptoms	D>V	V>D	D>V	D>V	V>D	V>D	D>V	V>D	D>V	V>D	D>V
Incubation period, average (hr)	5.8	4.0	3.5	4.5	6.0	3.8	10.0	4.1	3.6	5.0	2.0
Laboratory test											
Food	(-)	*Kudoa*	(-)	(-)	(-)	*Kudoa*	(-)	(-)	*Kudoa*	*Kudoa*	(-)
Environment	(-)	(-)	(-)	(-)	Problem in sanitation	(-)	(-)	(-)	(-)	(-)	(-)
Food preparer	(-)	(-)	(-)	(-)	(-)	(-)	(-)	(-)	(-)	(-)	(-)
Patient (no. of positive)	*Kudoa* (6)	*Kudoa* (8)	*Kudoa* (1)	*Kudoa* (2)	*Kudoa* (1) *B. cereus* (1)	*Kudoa* (1)	*Kudoa* (2)	*Kudoa* (3)	(-)	*Kudoa* (6)	*Kudoa* (2), but *Kudoa* in healthy (1)

V, vomiting; D, diarrhea.

터 12월까지 연중 검출되는 것으로 보고하였다. 그러므로 해당 식중독의 연중 발생률 변동에 대하여 장마철이나 한여름에는 광어회 섭취를 기피하게 되는 판매량 변동과 관련성 또한 고려해 보아야 한다. 임상적 증상은 세 병원체 모두 설사나 구토를 주소로 6시간 이내의 잠복기를 가지고, 24시간 이내에 증상 소실되는 동일한 특징을 보였다. 황색포도알균은 인간의 피부나 코점막에 상재하고, 바실루스 세레우스는 토양이나 자연계에 널리 분포하는 것으로 알려져 있다. 쿠도아충의 자연사는 잘 알려져 있지 않지만, 지금까지 밝혀진 바로는 광어에서 기생하는 것으로 알려져 있다[13]. 주된 감염 경로는 황색포도알균은 육류 및 유제품 등 매우 다양한 경로로 발생 가능하며, 바실루스 세레우스는 밥이나 육류 등을 통해 발생 가능하다. 칠성쿠도아충은 현재 광어 섭취 후에만 발생하는 것으로 되어 있다.

2. 칠성쿠도아충 식중독 사례 고찰

2015년 발생한 쿠도아충 식중독 개별 사례 11례에 대하여 요약해보았다(Table 2). 전체 11예 중 광어회만 섭취한 경우는 2예였고, 나머지 9예는 우럭, 조개류 등이 섞여 있는 모듬회 또는 초밥류 내에 광어가 포함되어 있었다. 11예 중 설사가 구토보다 우세하였던 경우는 6예, 구토가 우세하였던 경우는 5예였다. 평균 잠복기는 2-10시간이었다. 실험실적 결과에서 10예는 환례 검체에서 쿠도아충이 검출되었고, 1예(사례 9)는 환례에서는 쿠도아충이 검출되지 않고 보존식에서 검출되었다. 사례 5에서는 환례 검체에서 쿠도아충과 함께 바실루스 세레우스가 함께 검출되었고, 위생 상 문제점이 발견되었다. 사례 11에서는 환례 2명의 검체에서 쿠도아충이 검출되었지만, 증상이 없던 대조군 1예에서도 쿠도아충이 검출되었다.

3. 식중독 원인균 규명 현황

최근 3년(2013-2015년도)의 수인성 및 식품매개질환의 원인 파악 현황을 살펴보았다(Table 3). 원인 병원체 불명이 36-51%, 식중독 유발 감염원(음식 또는 식수) 불명이 75-81% 비율(2015년도 미보고)로 보고되었다. 밝혀진 원인균 중 겨울철의 대표적인 소화기계 감염증인 노로바이러스(16-19%)가 가장 높게 보고되었다. 뒤이어 병원성 대장균(6-10%), 클로스트리듐 퍼프린겐스(3-11%), 살모넬라(2-7%)가 식중독 원인체로 규명되었다. 황색포도알균증은 3년간 총 26건으로 전체의 2.4% (26/1,092)를 차지하였고, 바실루스 세레우스는 전체의 2.2% (24/1,092)를 차지하였다. 2015년을 기준으로 쿠도아충 식중독 사례가 총 11예이므로 전체 2015년 식중독 사례들 중 2.6%이고 5번째로 흔하게 관찰되는 식중독 원인이 된다.

Table 3. Annual trends in cause of food poisoning[1]

Rank	2015		2014		2013	
	Total	422	Total	409	Total	261
	Unknown	215 (51)	Unknown	186 (45)	Unknown	94 (36)
1	Norovirus	79 (19)	Norovirus	48 (12)	Norovirus	42 (16)
2	*E. coli*	26 (6)	*E. coli*	41 (10)	*C. perfringens*	30 (11)
3	*Campylobacter*	22 (5)	*C. perfringens*	29 (7)	*E. coli*	19 (7)
4	*C. perfringens*	14 (3)	Salmonellosis	28 (7)	Salmonellosis	12 (5)
5	Salmonellosis	10 (2)	*Campylobacter*	23 (6)	*Shigella* spp.	8 (3)
6	*S. aureus*	8 (2)	*S. aureus*	13 (3)	*B. cereus*	8 (3)
7	*B. cereus*	6 (1)	*B. cereus*	10 (2)	*V. parahaemolyticus*	8 (3)
8	*V. parahaemolyticus*	3 (1)	*V. parahaemolyticus*	6 (1)	*S. aureus*	5 (2)

Values are presented as number (%).
[1]Data from Korea Centers for Disease Control and Prevention. Epidemiological investigation of infectious diseases in Korea, annual report from 2013 to 2015 [7-9].

고 찰

칠성쿠도아충은 광어 취식 후 발생하는 식중독의 강력한 원인으로 최근 제시되었고 식중독 역학조사에서 광어가 식단에 포함되어 있을 시 쿠도아 원충 검사가 권장되고 있다. Song et al. [2]은 양식 육성어의 4.9%에서 칠성쿠도아충이 검출됨을 보고하였고, 광어회가 국민적 기호식품임을 감안하면 상당한 양의 칠성쿠도아충이 포함된 광어가 소비됨을 추측해 볼 수 있다. 칠성쿠도아충 식중독의 특징은 조리자의 주의 여부와 상관 없이 양식 육성어 공급 시에 이미 쿠도아충 감염 여부가 결정되어 있는 것이므로, 양식장 단계에서 쿠도아충 감염을 막는 것이 유일한 예방책일 것이다. 또한 현재까지 칠성쿠도아충은 광어에서만 발견되는 것으로 되어 있어, 타 식중독 역학조사와 다른 점은 환례 검체에서 쿠도아충 DNA가 검출되고 1일 이내 광어 취식력이 있다면 다른 보존식이나 환경검체 검사 결과에 관계 없이 쿠도아충으로 인한 식중독으로 결론 내려질 수 있다는 것이다.

하지만 위와 같은 결론을 내리기에는 광어쿠도아충이 식중독의 원인으로서의 병원성 여부가 명확하지 않아 성급하다고 생각된다. 인체에서의 병원성 여부를 증명하기 어려운 상황에서 동물실험 결과가 상충됨은 이미 Chung et al. [6]에 의해 논의된 바 있다. 식중독 환례의 검체에서 쿠도아충이 발견되었기 때문에 식중독병원체로 추정하기에는, 소화기계 증상을 일으키지 않았던 mice에서도 쿠도아충 DNA가 여전히 검출됨을 증명한 Ahn et al. [14]의 결과가 상충하게 된다. 이를 근거로 한다면 쿠도아충이 포함된 광어를 섭취한 경우 식중독 발생 여부와 관계 없이 대변에서 쿠도아충의 DNA가 검출될 수 있음을 가정해 볼 수 있다. 결과 1에서 살펴보았던 총 11예 중에서도 사례 11에서 증상이 없었던 대조군의 검체에서 쿠도아충이 발견되었음이 보고되었다. 그러므로 식중독 환례 검체에서의 쿠도아충 DNA가 검출 여부만으로 식중독 발생의 기여도를 판정하기에는 한계가 있다.

그렇다면 쿠도아충이 포함된 광어를 취식하고 왜 식중독이 발생하는 것인가? 저자는 황색포도알균이나 바실루스 세레우스에 의한 식중독이 쿠도아충에 의한 식중독으로 오인 혹은 동시에 혼재할 가능성을 제시해본다. 결과 2에서 살펴 보았듯이 쿠도아충 식중독과 위 두 병원체에 의한 식중독은 광어회 섭취력만 제외하면 역학적 및 임상적 특징이 매우 유사해 구분하기에 어려움이 따른다. 또한 결과 3에서 보았듯이 황색포도알균이나 바실루스 세레우스는 가장 흔한 식중독 원인임에도 불구하고, 각각 최근 3년간 전체 식중독의 2% 정도만 차지하는 것으로 되어 있다. 2015년을 기준으로 하면 오히려 쿠도아충에 의한 식중독보다 숫자가 작은 수준이다. 전체 식중독의 절반에서 원인병원체를 밝혀내지 못한 것을 생각해보면, 황색포도알균이나 바실루스 세레우스 식중독이 제대로 증명되지 못한 사례의 비율 또한 높을 것이며, 보고된 발생률보다 실제 발생률은 훨씬 높을 것으로 생각된다. 불명으로 처리된 황색포도알균이나 바실루스 세레우스 식중독이 쿠도아충 식중독으로 오인될 가능성 또한 존재하는 것이다. 결과 1의 사례 5에서는 환례 검체에서 쿠도아충이 1예 검출되었지만 동시에 바실루스 세레우스가 1예 검출된 것으로 보고되었다. 쿠도아충의 병원성이 불명확하다고 생각한다면 사례 5는 바실루스 세레우스에 의한 식중독으로 결론내릴 수도 있을 것이다.

이와 같은 고민의 주원인은 황색포도알균이나 바실루스 세레우스 식중독의 원인체 규명이 다른 식중독 원인보다 어렵다는 데 기인한다. 황색포도알균이나 바실루스 세레우스 식중독을 증명하는 데 있어 역학적 연관성이 있는 음식에서 균증식 혹은 장독소를 증명하고, 독소형 식중독에 합당한 임상증상(짧은 잠복기, 가벼운 소화기계 증상)을 보인다면 충분히 역학적 추정이 가능할 것이다. 하지만 일반적으로 발생하는 식중독에서 임상양상으로 독소형 식중독이 의심되더라도, 보존식이 있는 사례가 매우 드물고, 환례의 대변 배양을 할 수 있는 경우도 극히 제한되어서 실험적인 근거로 증명되는 경우는 매우 소수이고 대부분이 원인 불명

으로 분류되게 된다[10]. 또한 독소형 식중독의 원인음식이 재조리되어 제공된 경우, 원인병원균은 사라지고 열저항성이 있는 장독소나 포자만 남아 식중독을 일으키기 때문에 실험실적으로 증명하기 매우 어렵다.

식중독 역학조사에 있어 원인을 규명한다는 것은 원인 제공 업체에 대한 행정적 처분이 따르고, 미래의 보건 정책에도 영향을 줄 수 있기에 매우 신중하여야 하겠다. 칠성쿠도아충이 식중독을 일으켰다는 주장이 설득력을 얻기 위해선 황색포도알균이나 바실루스 세레우스 식중독과 같은 형태의 규명률을 높이기 위한 꾸준한 노력과 칠성쿠도아충의 병인성에 대한 연구가 계속해서 보강되어야 하겠다.

참고문헌

1. Korea Centers for Disease Control and Prevention. Case reports of Kudoa food poisoning in 2015; 2016 [cited 2016 Dec 11]. Available from: http://cdc.go.kr/CDC/notice/CdcKrTogether0302.jsp?menuIds=HOME001-MNU1154-MNU0005-MNU0088&cid=71877 (Korean).
2. Song JY, Choi JH, Choi HS, Jung SH, Park MA. Monitoring of Kudoa septempunctata in cultured olive flounder and wild fish in Jeju Island during 2012. J Fish Pathol 2013;26:129-137 (Korean).
3. Kawai T, Sekizuka T, Yahata Y, Kuroda M, Kumeda Y, Iijima Y, et al. Identification of Kudoa septempunctata as the causative agent of novel food poisoning outbreaks in Japan by consumption of Paralichthys olivaceus in raw fish. Clin Infect Dis 2012;54:1046-1052.
4. Yoshiko Sugita-Konishi Y, Sato H, Ohnishi T. Novel foodborne disease associated with consumption of raw fish, olive flounder (Paralichthys olivaceus). Food Saf 2014;2:141-150.
5. Jang Y, Ahn M, Bang H, Kang B. Effects of Kudoa septempunctata genotype ST3 isolate from Korea on ddY suckling mice. Parasite 2016;23:18.
6. Chung YB, Bae JM. Is there evidence that Kudoa septempunctata can cause an outbreak of acute food poisoning? Epidemiol Health 2017;39:e2017004.
7. Korea Centers for Disease Control and Prevention. Epidemiological investigation of infectious diseases in Korea, annual report 2013; 2014 [cited 2014 Apr 21]. Available from: http://www.cdc.go.kr/CDC/contents/CdcKrContentLink.jsp?fid=43&cid=26002&ctype=1 (Korean).
8. Korea Centers for Disease Control and Prevention. Epidemiological investigation of infectious diseases in Korea, annual report 2014; 2016 [cited 2016 Apr 14]. Available from: http://www.cdc.go.kr/CDC/contents/CdcKrContentLink.jsp?fid=43&cid=67789&ctype=1 (Korean).
9. Korea Centers for Disease Control and Prevention. Epidemiological investigation of infectious diseases in Korea, annual report 2015; 2016 [cited 2016 Nov 16]. Available from: http://www.cdc.go.kr/CDC/contents/CdcKrContentLink.jsp?fid=51&cid=71877&ctype=1 (Korean).
10. Juneja VK, Sofos JN. Pathogens and toxins in foods: challenges and interventions. Washington, DC: ASM Press; 2010, p. 1-130.
11. Hennekinne JA, De Buyser ML, Dragacci S. Staphylococcus aureus and its food poisoning toxins: characterization and outbreak investigation. FEMS Microbiol Rev 2012;36:815-836.
12. Granum PE, Lund T. Bacillus cereus and its food poisoning toxins. FEMS Microbiol Lett 1997;157:223-228.
13. Kasai A, Li YC, Mafie E, Sato H. New host records of monacanthid fish for three Kudoa spp. (K. septempunctata, K. thyrsites, and K. shiomitsui) prevalent in the olive flounder (Paralichthys olivaceus), with the description of K. parathyrsites n. sp. from a black scraper (Thamnaconus modestus). Parasitol Res 2016;115:2741-2755.
14. Ahn M, Woo H, Kang B, Jang Y, Shin T. Effect of oral administration of Kudoa septempunctata genotype ST3 in adult BALB/c mice. Parasite 2015;22:35.
